# Enhancing triapine treatment: strategies for dose optimization and methemoglobin level mitigation

**DOI:** 10.1007/s00280-026-04898-6

**Published:** 2026-05-20

**Authors:** Heekyung Lee, Allison Dunn, Sarah E. Taylor, Aman Chauhan, S. Percy Ivy, Jogarao Gobburu, Jan H. Beumer

**Affiliations:** 1 Center for Translational Medicine, University of Maryland School of Pharmacy, 20 North Pine Street, Baltimore, MD 21201 USA; 2 Division of Gynecologic Oncology, Department of Obstetrics, Gynecology and Reproductive Sciences, University of Pittsburgh School of Medicine, Pittsburgh, PA USA; 3 Sylvester Comprehensive Cancer Center, University of Miami, Miami, FL USA; 4 Investigational Drug Branch, Cancer Therapy Evaluation Program, Division of Cancer Treatment and Diagnosis, National Cancer Institute, Bethesda, MD USA; 5 Cancer Therapeutics Program, UPMC Hillman Cancer Centre, Pittsburgh, PA USA; 6 Department of Oncology, Johns Hopkins University School of Medicine and Johns Hopkins Sidney Kimmel Cancer Center, 1650 Orleans St, Room 1M52, Baltimore, MD 21287 USA

**Keywords:** Triapine, Pharmacokinetics, Pharmacodynamics, Methemoglobin, Radiosensitizer

## Abstract

**Purpose:**

Triapine is a potent small-molecule ribonucleotide reductase inhibitor investigated in combination with radiation and/or chemotherapy for the treatment of advanced stage solid cancers. The aim of this study is to develop a population pharmacokinetic-pharmacodynamic (PK/PD) model for triapine to describe the PK parameters, the effect of smoking on exposure, and the relationship between methemoglobin concentrations and exposure.

**Methods:**

A total of 36 patients with advanced stage cervical or neuroendocrine cancers from two phase I studies were included in the population PK/PD model building. Triapine and methemoblogin plasma concentrations were sampled over an 8-hr or 24-hour period. Data were analyzed by a nonlinear mixed-effects modelling approach. Simulations were performed to optimize the dosing strategy for oral triapine.

**Results:**

A two-compartment model with two-transit compartment Erlang absorption and first-order elimination best described the PK of triapine, and an effect compartment model best described the PD effect of triapine on methemoglobin concentrations. The final model described triapine PK/PD well, and a 38% increase in triapine clearance was estimated due to smoking. Simulations suggest that dose adjustments may be necessary, as increasing the oral dose for smokers from 100 to 125 mg resulted in exposures matching those observed in nonsmokers.

**Conclusion:**

This study provides a quantitative model characterizing the relationship between triapine exposure and methemoglobin concentrations. The developed PK/PD model can be used to optimize the dosing regimen for oral triapine, illustrating how population PK/PD modeling can inform decision-making throughout the triapine drug development lifecycle.

**Supplementary Information:**

The online version contains supplementary material available at 10.1007/s00280-026-04898-6.

## Introduction

Radiation and/or chemotherapy are primary modalities used in cancer treatment [1, 2]. To enhance the sensitivity of cancer cells to radiation or chemoradiation therapy, radiosensitizers are used in conjunction to improve the treatment outcome [3, 4]. Radiosensitizers increase the damage to cancer cells when exposed to radiation, often by enhancing DNA damage or inhibiting repair mechanisms. Ribonucleotide reductase (RNR) is an essential enzyme that converts ribonucleotides into deoxyribonucleotides, which are the key building blocks for DNA synthesis and repair [5]. Inhibiting this rate limiting enzyme can disrupt DNA repair mechanisms, increase radiation-induced DNA damage, and promote synergistic effects with radiation therapy. Thus, RNR inhibitors have been developed as potential radiosensitizers [6, 7].

Triapine (3-aminopyridine-2-carboxaldehyde thiosemicarbazone) is a potent small-molecule RNR inhibitor [6–8]. Clinical phase 1 and 2 trials combining triapine with standard radiation or chemoradiation therapy for the treatment of various types of advanced stage solid cancers have shown promising results [9–18], and although a recent phase 3 trial read out negative [19], other trials remain ongoing. While triapine treatment is generally safe and well tolerated, studies have shown that triapine can induce methemoglobin (mHb) formation and hypoxia in patients [17, 20–22]. Furthermore, in vitro studies have shown that triapine is metabolized by cytochrome P450 (CYP) enzymes, with CYP1A2 accounting for most of the metabolism of triapine [23]. Activity of CYP1A2 can be induced by tobacco smoke [24]. However, the effect of smoking on triapine exposure using a population PK model has not been previously examined. A comprehensive understanding of the exposure-response relationship is crucial for optimizing treatment and guiding decision-making in triapine drug development.

The aim of this study was to develop a population pharmacokinetic (PK) and pharmacodynamic (PD) model to characterize the disposition of triapine, the effect of smoking on exposure, and the effect on mHb concentration with exposure. The developed PK/PD model was then leveraged to optimize dosing strategy for oral triapine.

## Methods

### Participants and study design

Data from two phase I trials (NCT02595879 and NCT04234568) were analyzed. Each study protocol was approved by their respective investigational review board or human subjects committee. Study NCT02595879 (NCI 9892) was a phase I dose-escalation bioavailability study of oral triapine in combination with concurrent chemoradiation for locally advanced cervical cancer and vaginal cancer [25]. Patients were given once a day oral triapine (100 mg or 150 mg) 5 days a week for 5 weeks, except on day 1 (and day 11 for *n* = 5 patients) where 50 mg IV triapine (2-hour infusion) was administered to assess oral bioavailability. PK samples were collected on days 1 and 8 (and days 4 and 11 for *n* = 5 patients) at the following time points: prior to dose, 0.5, 1, 1.5, 2, 2.5, 3, 4, 6, 8, and 24 h after dose. Methemoglobin (mHb) was determined on PK days at the following time points: prior to triapine dose, 4 and 24 h after dose. Study NCT04234568 (NCI 10388) was a multicenter phase I trial of triapine and Lutetium Lu 177 Dotatate in combination for well-differentiated somatostatin receptor-positive gastroenteropancreatic neuroendocrine tumors (GEP-NETs). Patients were given once a day oral triapine (100 mg or 150 mg) on days 1–14 of an 8-week cycle. PK and mHb samples were collected on day 9 at the following time points: prior to dose, 0.5, 1, 1.5, 2, 3, 4, 6, and 8 h after dose. Individual concentration-time curves are shown in Figure S1.

### Bioanalysis

Plasma concentrations of triapine were evaluated using a validated liquid-chromatography, mass spectrometry (LC-MS/MS) assay with a lower limit of quantification (LLQ) at 3 µg/L as previously described [26]. Briefly, protein precipitation supernatant was separated by chromatography using a Shodex ODP2 column and an isocratic acetonitrile-water mobile phase with 10% ammonium acetate. Detection was performed using an ABI 4000 mass spectrometer employing electrospray positive mode ionization. The assay was linear from 3 to 3000 µg/L, with an accuracy range of 97.1–103.1% and precision of < 7.4% CV. Methemoglobin concentrations were measured in the peripheral venous blood per local standard of care. Briefly, methemoglobin concentrations were quantified as the fraction of total hemoglobin on a venous blood gas analyzer equipped with multi-wavelength co-oximetry.

### Estimation methods

PK observations for model development include triapine plasma concentration, and PD observations include mHb concentrations. A sequential population PK/PD modeling approach was employed using first-order conditional estimation (FOCE) with eta-epsilon interaction method to fit the observed data. Typical PK/PD parameters, the between-subject variability (BSV), and the within-subject variability (residual unexplained variability) parameters were estimated. BSV was incorporated as exponential random error models on model parameters, assuming a log-normal distribution:

$$\:{\mathrm{P}}_{\mathrm{i}}={\mathrm{P}}_{\mathrm{p}\mathrm{o}\mathrm{p}}\:\mathrm{x}\:{\mathrm{e}}^{{\upeta\:}\mathrm{i}}$$

where P_i_ is the individual PK/PD parameter estimate for subject i, P_pop_ is the typical population value for the PK/PD parameter, and η_i_ is the individual random effect estimate representing the deviation from P_pop_ for subject i, assumed from a normal distribution with a mean of zero and variance ω^2^. For interpretation purposes, BSV was expressed as percent coefficient of variation (%CV), which was calculated as $\:\sqrt{{e}^{\omega\:2}-1}$ x 100%.

For bioavailability (F) parameter, BSV was incorporated using a logit-transformation, assuming a logit-normal distribution:

$$\:{\mathrm{F}}_{\mathrm{i}}={\mathrm{l}\mathrm{o}\mathrm{g}\mathrm{i}\mathrm{s}\mathrm{t}\mathrm{i}\mathrm{c}\left({\upeta\:}\mathrm{F}\right),\:\mathrm{w}\mathrm{h}\mathrm{e}\mathrm{r}\mathrm{e}\:{\upeta\:}\mathrm{F}\:\sim\:\mathrm{N}\mathrm{o}\mathrm{r}\mathrm{m}\mathrm{a}\mathrm{l}\:\left(\mathrm{l}\mathrm{o}\mathrm{g}\mathrm{i}\mathrm{t}\right(\mathrm{F}}_{pop}),{\omega\:}^{2})\:$$

where F_i_ is the individual F parameter estimate for subject i, F_pop_ is the typical population value for the F parameter, and η_F_ is the individual random effect estimate representing the deviation from F_pop_ for subject i. The %CV of the parameter was calculated using the delta method [27, 28], approximated as (1-F_pop_) * sqrt(ω^2^).

Combined (additive + proportional) residual error model best described the variability in the difference between the individual predictions and observations that remained unexplained.

### Population pharmacokinetic modeling

A stepwise approach with sequential addition of peripheral compartments was used to determine the structural model of triapine. A 2-compartment model with a 2-transit compartment Erlang absorption and a first order elimination was selected to best describe the observed triapine PK. Code for PK model is in Methods S1.

There were 605 PK samples from all subjects, of which 32 were below the lower limit of quantification (LLOQ) at the 24-hour time point. For the main analysis, LLOQ observations were omitted in the PK modeling using the M1 method [29]. To evaluate the impact of omitting LLOQ observations, the M3 method [29], which retains LLOQ observations and treats them as censored, was tested for comparison and yielded similar parameter estimates (Table S1).

*Covariate PK Model*. Once the structural and statistical models were developed, the effect of individual characteristics and pertinent extrinsic factors (covariates) was explored for their ability to explain variability in triapine PK. Continuous covariates explored in this analysis included weight and age; while categorical covariates explored included sex and smoking status. Covariates were added using the forward selection method. A 3.84 decrease in the objective function values (OFV) for 1 degree of freedom was considered statistically significant (*p* < 0.05). Adding weight and smoking status significantly reduced the OFV, but sex and age had no significant effect. Only weight and smoking status were included as covariates in the final model. The effect of actual body weight was introduced in the model using allometric scaling:

$$\:{\mathrm{P}}_{\mathrm{i}}={\mathrm{P}}_{\mathrm{p}\mathrm{o}\mathrm{p}}\:\mathrm{x}\:\:{\left(\frac{\mathrm{W}\mathrm{T}\mathrm{i}}{70}\right)}^{{\Theta\:}}\:{\mathrm{x}\:\mathrm{e}}^{{\upeta\:}\mathrm{i}}$$

where P_i_ is the PK parameter value for subject i; P_pop_ is the typical population value for the PK parameter for a reference subject weighing 70 kg; WTi is the weight of the subject i; Θ is the allometric scaling power that was fixed to 0.75 for all clearance terms and fixed to 1 for all volume terms; η_i_ is the individual deviation from P_pop_.

### Population Pharmacodynamic Modeling

An effect compartment model was selected to characterize the effect of triapine exposure on the methemoglobin (mHb) concentrations. Code for PD model is in Methods S2. Effect is driven by the concentration in the effect compartment, which is delayed relative to plasma concentration by a first-order effect compartment rate constant k_e0_ [30]. The rate constant ke0 was defined as:

$$\:\frac{{\mathrm{d}\mathrm{C}}_{\mathrm{e}\mathrm{f}\mathrm{f}}}{\mathrm{d}\mathrm{t}}=\:{\mathrm{k}}_{\mathrm{e}0}\mathrm{*}({\mathrm{C}}_{\mathrm{p}}-\:{\mathrm{C}}_{\mathrm{e}\mathrm{f}\mathrm{f}})$$

where C_eff_ is the concentration in the effect compartment and C_p_ is the concentration in plasma. The triapine effect on mHb concentration was described as:

$$\:\mathrm{E}=\:{\mathrm{E}}_{\mathrm{b}\mathrm{a}\mathrm{s}\mathrm{e}}\mathrm{*} \left (1+\:\frac{{\mathrm{E}}_{\mathrm{m}\mathrm{a}\mathrm{x}}\mathrm{*}\:{\mathrm{C}}_{\mathrm{e}\mathrm{f}\mathrm{f}}^{\mathrm{h}}}{{\mathrm{E}\mathrm{C}}_{50}^{\mathrm{h}}+\:{\mathrm{C}}_{\mathrm{e}\mathrm{f}\mathrm{f}}^{\mathrm{h}}}\right)$$

where E is the effect; E_base_ is the effect at baseline; E_max_ is the maximum effect; EC_50_ is the concentration that elicits 50% maximal effect; C_eff_ is the concentration in the effect compartment; h is the Hill coefficient.

### Final model qualification

A nonparametric bootstrap simulation was performed to evaluate the stability and robustness of the final sequential PK/PD model. A total of 500 datasets were generated by sampling individuals with replacement from the original dataset. The final population PK/PD model was fitted to each generated dataset, and the median and 95% confidence intervals (CI) of parameter estimates were compared with the final population PK/PD model. A prediction-corrected visual predictive check (pcVPC) and quantitative predictive check (QPC) were performed to additionally evaluate the validity of the model. For pcVPC, a total of 500 replicate simulations were performed for the same trial design that assumed typical values of model parameters and variability estimates determined by the final model. The 10th, 50th, and 90th percentiles of the observed concentrations were assessed graphically by comparing with the 95% CI of the corresponding percentiles of the simulated data. For QPC, a total of 1000 replication simulations were performed. Each iteration corresponds to one full simulated population. For each iteration, AUC_(0−∞)_ and C_max_ were calculated using noncompartmental analysis (NCA), as was done for the observed data. The median value for AUC_(0−∞)_ and C_max_ for each simulated population were then compared graphically to the corresponding median value of the observed data.

### Simulations for dosing strategy

There were two parts to the simulations for dosing strategy: (1) The goal of the first simulation was to propose an appropriate dose such that 90% or more of the population reach the target concentration (C_max_ above 200 µg/L [18]) and stay below the mHb threshold (< 10% [31]) for smokers and nonsmokers. Parametric simulations were performed separately for 1000 smokers and 1000 nonsmokers with a weight range of 40 to 100 kg, reflecting the actual body weight range in the observed dataset. Since smokers have a 38% increase in CL (Table 2), smokers were simulated with 125 mg and nonsmokers were simulated with 100 mg of oral triapine. (2) The goal of the second simulation was to assess how the between occasion variability (BOV) applied on the bioavailability parameter affects the percentage of the population that exceeds the mHb threshold when multiple doses are administered, as would occur in a typical clinical situation. BOV was not estimated in the final PK model, but “artificial” BOV ranging from 0% − 60% was added with BSV on bioavailability parameter for simulation purposes. A population of 1000 subjects were simulated with 100 mg of oral triapine given once daily for 28 days with BOV ranging from 0% − 60%.

### Software

The analysis was performed using nonlinear mixed-effects modeling in Pumas (version 2.5.1, Pumas AI, Dover, DE, https://pumas.ai/). Visual inspections were performed in R (version 4.3.1, R Core Team, 2023, Vienna, Austria, https://www.R-project.org/).

## Results

### Study population

A total of 36 patients from two phase I studies (NCT02595879 and NCT04234568) were analyzed to develop the final PK/PD model. There were 573 quantifiable plasma concentrations from all 36 patients for the PK model development and 252 quantifiable mHb concentrations from 33 patients for the PD model development. The relevant patient demographics are summarized in Table 1.

**Table 1:** Summary Statistics of Patient Demographics

	NCT02595879 NCI 9892 (*n* = 20)	NCT04234568 NCI 10,388 (*n* = 16)
Number of subjects per dose group	Inf = 50 mg (*n* = 20)	
	PO = 100 mg (*n* = 16)	PO = 100 mg (*n* = 3)
	PO = 150 mg (*n* = 4)	PO = 150 mg (*n* = 13)
Sex	Female = 20	Female = 4
		Male = 12
**Age (years)**		
Mean (SD)	50.1 (13.6)	51.9 (10.9)
Median	52.5	49.0
[Min, Max]	[23, 71]	[37, 74]
Weight (kg)		
Mean (SD)	77.4 (14.1)	[53.8, 138.4]
Median	78.6	[53.8, 138.4]
[Min, Max]	[41.8, 103.9]	[53.8, 138.4]
Smoking Status	No = 14	No = 16
	Yes = 6	
Race	White = 18	White = 15
	Asian = 1	Black/African Am = 1
	Not Reported = 1	Liver = 2
Cancer type	Cervical = 18	Large Intestine = 1
	Vaginal = 2	Small Intestine = 3
		Colon = 2
		Pancreas = 8

Inf, infusion; PO, oral; SD, standard deviation

### Population pharmacokinetic modeling

**Fig. 1:**
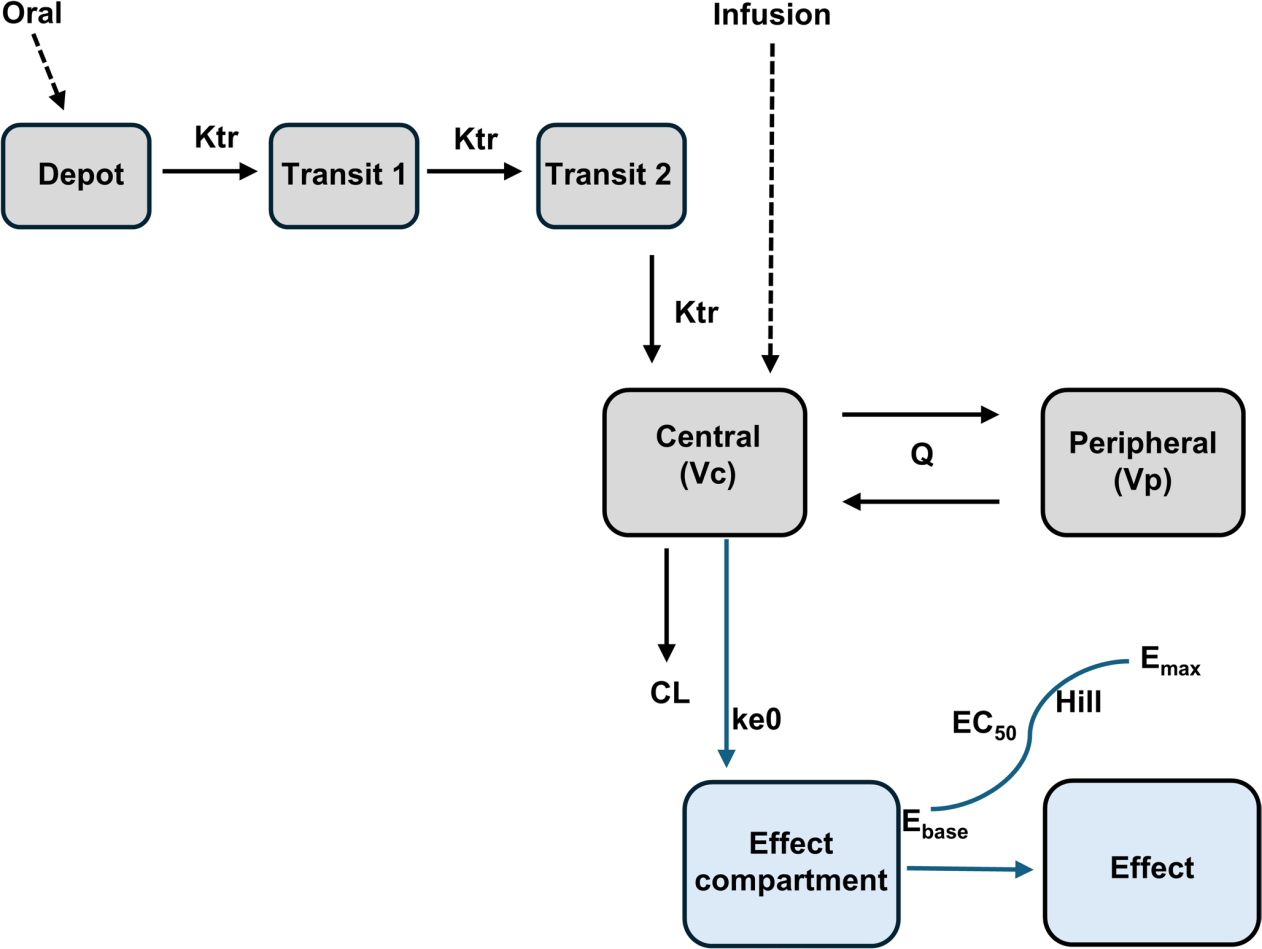
Schematic of Structural PK/PD Model. The structural PK model is a two-compartment model with two-transit Erlang absorption and first-order elimination, as indicated in gray color. The PD model is an effect compartment model, as indicated in blue color. Ktr, transit rate constant; CL, clearance; Vc, central volume of distribution; Vp, peripheral volume of distribution; Q, intercompartmental clearance, ke0, first-order effect compartment rate constant; E_base_, baseline steady state of the effect; E_max_, maximum effect; Hill, Hill coefficient; EC50, half-maximal effective concentration

**Fig. 2:**
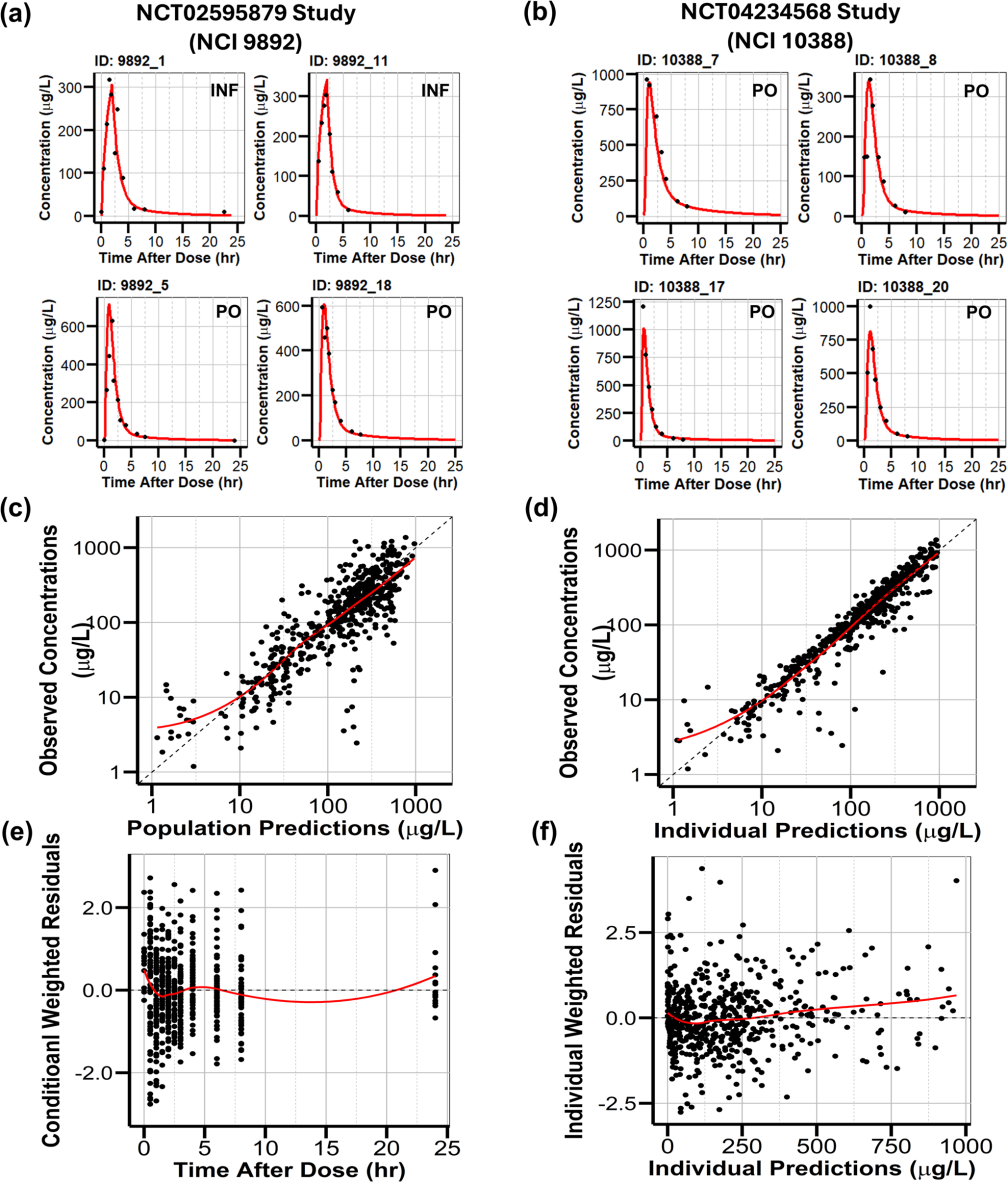
Individual Fits and Goodness-of-Fit Plots for the final PK model. Individual predictions of representative subjects from (**a**) NCT02595879 study and (**b**) NCT04234568 study. Black dots represent the observed triapine concentrations and red lines represent the predicted fits. Goodness-of-fit plots for (**c**) the population predicted versus the observed concentration, (**d**) the individual predicted versus the observed concentration, (**e**) the conditional weighted residual plot versus time, and (**f**) the individual weighted residual plot versus the individual predictions. The red line depicts the loess fit for the data

The structural model that best described triapine PK was a two-transit compartment Erlang absorption with a two-compartment distribution model with first-order elimination from the central compartment (Fig. 1). A combination additive and proportional residual error model best described the residual error. The BSV was estimated for Ktr, CL, Vc, and F. The BSV was not estimated for Q and Vp due to over-parameterization and inability to minimize when included. Plots of the individual predictions for the observed concentrations demonstrated a good fit (Fig. 2A, B).

The effect of smoking status on CL was introduced into the model as a multiplicative effect: $\:{\:\mathrm{P}}_{\mathrm{i}}={\mathrm{P}}_{\mathrm{p}\mathrm{o}\mathrm{p}}\:\mathrm{x}\:\left(1+{\upbeta\:}\mathrm{*}\mathrm{s}\mathrm{m}\mathrm{o}\mathrm{k}\mathrm{i}\mathrm{n}\mathrm{g}\:\mathrm{s}\mathrm{t}\mathrm{a}\mathrm{t}\mathrm{u}\mathrm{s}\right)\:\mathrm{x}\:{\mathrm{e}}^{{\upeta\:}\mathrm{i}}$

where P_i_ is the PK parameter value for subject i; P_pop_ is the typical population value for the PK parameter; β is the effect size of smoking; smoking status is a binary variable (0 = nonsmoker, 1 = active smoker); η_i_ is the individual deviation from P_pop_. The model estimated effect size of smoking on CL was 0.38, with a delta OFV of 5.06, suggesting that the smokers have 38% higher clearance compared to the nonsmokers.

The population PK parameter estimates from the final model are reported in Table 2. The typical transit absorption rate constant (Ktr) was estimated to be 1.0/h. The estimated parameters for a typical subject with a body weight of 70 kg are as follows: Cl = 51.8 L/h, Vc = 67.9 L, Q = 12.6 L/h, Vp = 65.5 L, and F = 0.76. A moderate shrinkage (33%) was observed in Vc and consequently diagnostics with individual Vc may be biased, but the shrinkage does not affect parameter estimation. Overall, the final PK model yielded physiologically plausible estimates for triapine disposition parameters.

**Table 2:** Final PK/PD Parameter Estimates

PK Parameters	Units	Final Model Estimate	Bootstrap (*N* = 500)^a^ 95% CI
Ktr	(1/h)	3.11	[2.63; 3.72]
CL^b^	(L/h)	51.8	[45.0; 60.2]
Vc^b^	(L)	67.9	[46.9; 75.9]
Q^b^	(L/h)	12.6	[10.2; 27.3]
Vp^b^	(L)	65.5	[28.9; 100]
F		0.76	[0.64; 0.88]
Effect_smoker		0.38	[0.19; 0.62]
**PD Parameters**			
ke0	(1/h)	0.36	[0.25; 0.42]
E_base_	(%)	0.95	[0.81; 1.09]
E_max_ (fixed)	(%)	100	
EC_50_	(mg/L)	2535	[1414; 4514]
Hill		1.63	[1.35; 2.07]
**Between-Subject Variability (BSV)**			
$\omega_ {Ktr}$	BSV (%CV)	47.8 [shrinkage: 8.0%]	[31.1; 63.5]
$\omega_ {CL}$	BSV (%CV)	28.8 [shrinkage: 12.3%]	[19.6; 36.9]
$\omega_{Vc}$	BSV (%CV)	22.2 [shrinkage: 33.0%]	[13.2; 32.3]
$\omega_{F}$	BSV (%CV)	29.5 [shrinkage: 17.4%]	[23.3; 34.6]
$\omega_{Ebase}$	BSV (%CV)	34.7 [shrinkage: 9.3%]	[24.3; 44.6]
$\omega_{EC50}$	BSV (%CV)	57.9 [shrinkage: 11.9%]	[33.5; 85.3]
**Residual Unexplained Variability (RUV)**			
$\sigma$PK_additive	($\mu$g/L)	3.94	[2.24; 5.22]
$\sigma$PK_proportional	(%CV)	33.2	[26.8; 39.2]
$\sigma$PD_additive	($\mu$g/L)	0.13	[0.01; 0.23]
$\sigma$PD_proportional	(%CV)	27.3	[18.2; 33.9]

Ktr, transit rate constant; CL, clearance; Vc, central volume of distribution; Q, intercompartmental clearance; Vp, peripheral volume of distribution; F, bioavailability; Effect_smoker, effect of smoking on CL; ke0, first-order effect compartment rate constant; E_base_, baseline steady state of the effect; E_max_, maximum effect; Hill, Hill coefficient; EC_50_, half-maximal effective concentration; σ_additive_, additive residual error; σ_proportional_, proportional residual error; CI, confidence interval

^a^Final model’s bootstrap estimates; 95% CI are reported as median and [2.5th, 97.5th percentiles] of the parameter estimates​

^b^Final model parameter estimates for population clearances and volumes of distribution are standardized to a 70 kg reference subject through allometric scaling

The final PK model provided a reasonable description of the data, as illustrated by the diagnostic goodness of fit (GOF) plots. The plots for the population predicted versus observed concentration (Fig. 2C) and the individual predicted versus observed concentration (Fig. 2D) revealed that observations were evenly distributed around the line of identity. The residual plots versus time (Fig. 2E) and versus individual predictions (Fig. 2F) did not demonstrate a trend around the zero line. These diagnostic plots all demonstrate the adequacy of the structural model.

### Population pharmacodynamic modeling

**Fig. 3:**
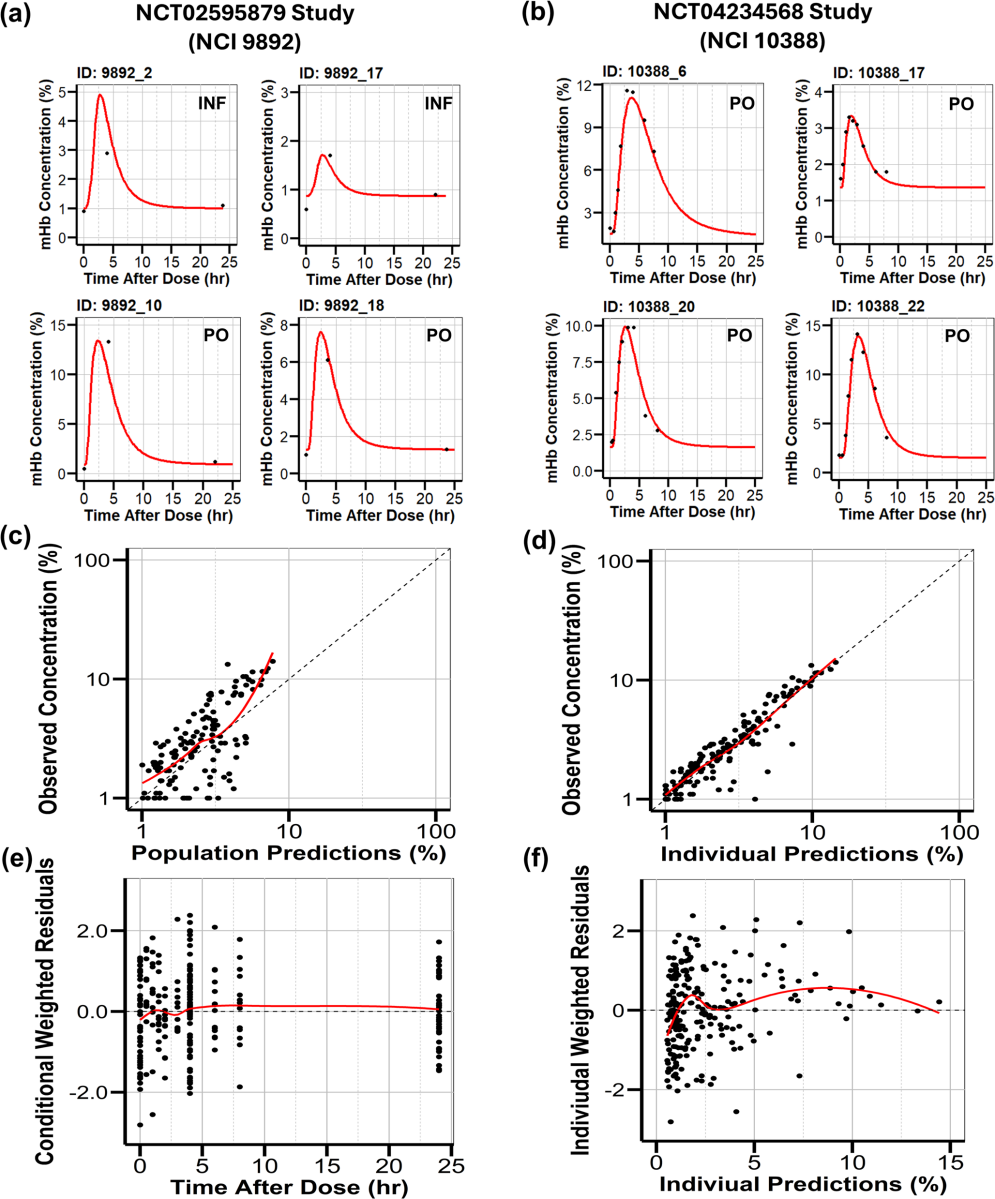
Individual Fits and Goodness-of-Fit Plots for the final PD model. Individual predictions of representative subjects from (**a**) NCT02595879 study with sparse mHb sampling data points (3 time points for each individual) and (**b**) NCT04234568 study with robust mHb sampling data points (9 time points for each individual). Black dots represent the observed mHb concentrations and red lines represent the predicted fits. Goodness-of-fit plots for (**c**) the population predicted versus the observed concentration, (**d**) the individual predicted versus the observed concentration, (**e**) the conditional weighted residual plot versus time, and (**f**) the individual weighted residual plot versus the individual predictions. The red line depicts the loess fit for the data

The individual predictions from the final PK model were used to inform the subsequent PD model. The relationship between triapine concentration and mHb concentration showed hysteresis, indicating there was a time delay between exposure to triapine and the subsequent mHb concentration response (Fig. S2). An effect compartment model reliably described this relationship (Fig. 1). Plots of the individual predictions for the observed mHb concentrations demonstrated a good fit (Fig. 3A, B). The estimated first-order effect compartment rate constant (ke0) was 0.36/h. The estimated baseline steady state of the effect (E_base_) was 0.95%. The predicted concentration of triapine producing 50% of the maximal effect (EC_50_) on mHb concentration was 2535 µg/L, suggesting low potency at the effect site and that typical plasma concentrations would produce only a modest effect. The maximum effect (E_max_) of triapine on mHb concentration was fixed at 100%. In the observed data, the highest observed mHb concentration was low (14.1%), with concentrations ranging from 1.0 to 14.1%. Since the observed mHb concentrations never approach saturation, the model could not reliably determine the top of the E_max_ curve, resulting in unrealistically high estimates. Thus, given the limited data, E_max_ was fixed at 100%, since physiologically E_max_ cannot exceed 100% of total hemoglobin oxidized. Estimation of EC_50_ and E_max_ from the available data may be limited and variable, and these results should be interpreted with caution. A combined additive and proportional residual error model most appropriately described the residual unexplained variability. The BSV was estimated for E_base_ and EC_50_. The population PD parameter estimates from the final model are reported in Table 2.

Overall, the PD model provided a reasonable description of the data, as demonstrated by the GOF plots. The plots of the population (Fig. 3C) and individual (Fig. 3D) predicted versus observed mHb concentrations were evenly distributed around the line of identity. The residual plots (Fig. 3E, F) did not demonstrate a trend around the zero line, further indicating the adequacy of the final structural PD model.

### Final PK/PD model qualification

**Fig. 4:**
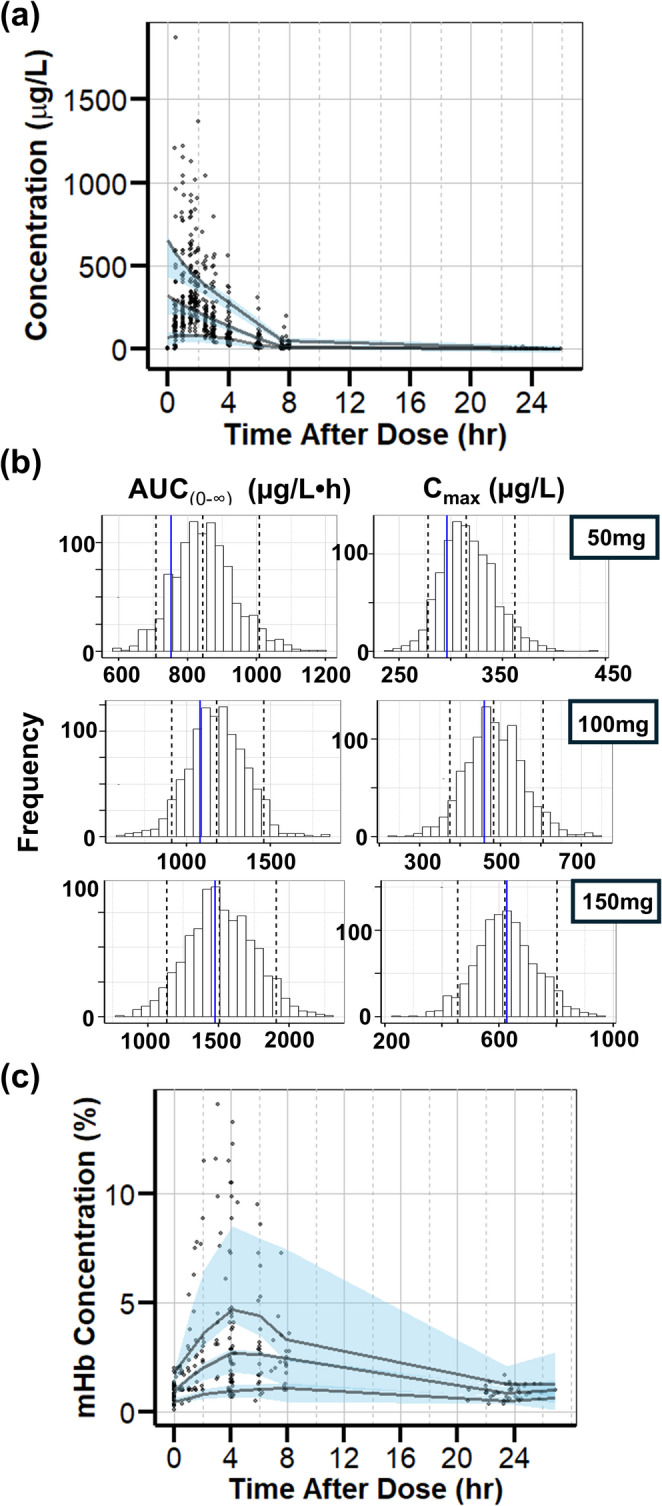
Final PK/PD Model Qualifications. (**a**) Prediction-corrected visual predictive check (pcVPC) for the developed PK model. Solid lines are the 10th, 50th, and 90th percentiles of the prediction-corrected observed data, and the shaded bands are the 95% CI of the corresponding percentiles of the simulated prediction-corrected data. Raw observed data points are plotted in black circles. (**b**) Quantitative predictive check for the developed PK model. The distribution shows the simulated 50th percentile values for AUC_(0−∞)_ and C_max_ for each dosage group. The blue line represents the 50th percentile values of the observed data. The dashed lines represent the 5th and 95th quantiles of the simulated data. (c) pcVPC for the developed PD model

Bootstrap simulations conducted to validate the final model further confirmed that the PK parameters and PD parameters were estimated with high precision, as shown by the resulting narrow confidence intervals (Table 2). The results of the pcVPC (Fig. 4A) for the final PK model and the pcVPC for the final PD model (Fig. 4C) demonstrated that the 10th, 50th, and 90th percentiles of observed concentrations properly align within the 95% CI of each percentile from simulated datasets, illustrating that the variability of the data was adequately captured. The results of the QPC for the final PK model (Fig. 4B) further showed that the 50th percentiles of observed AUC_(0−∞)_ and C_max_ for each dose group fall within the 95% CI of the simulated percentile values. These results all support a strong agreement between the predicted and observed concentrations, validating the stability and robustness of the final PK/PD model.

### Simulations for dosage strategy

Triapine is available in both IV and PO formulations. While most phase 1 and 2 studies have administered IV triapine [10, 11, 15, 17, 18, 20], receiving a 2-hour infusion multiple times a week can be an intensive regimen for patients. Oral dosing may be a more feasible option, potentially offering even greater clinical benefits with daily administration. Thus, simulations were performed to optimize dosing strategy for oral triapine.

**Fig. 5:**
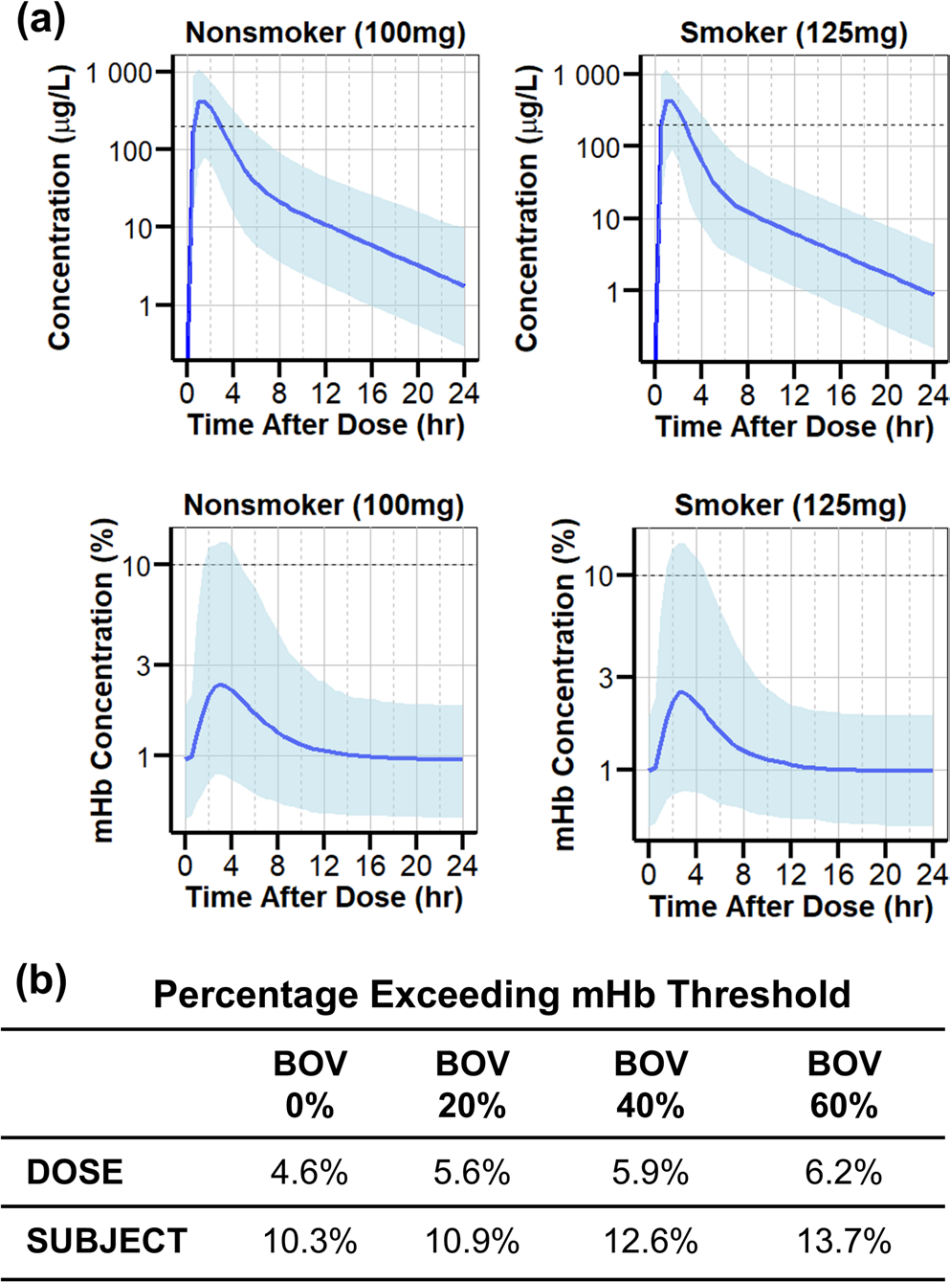
Simulations for Dosing Strategy. (**a**) Simulation with a single oral dose of 100 mg for nonsmokers and 125 mg for smokers showed that > 90% of the subjects achieved a C_max_ above the target concentration of 200 µg/L, with mHb concentrations below 10%. Dashed lines indicate the target threshold. Blue solid lines indicate 50th percentile and the shaded lines indicate 2.5th and 97.5th percentiles of the prediction intervals. (**b**) A population of 1000 subjects were simulated with 100 mg of oral triapine given once daily for 28 days (i.e., each subject has 28 doses). As %BOV increased, the percentage of dose (total number of doses from all subjects) and subject (total number of subjects) exceeding mHb concentration threshold increased

The aims of the simulations were two-fold. The first aim was to propose a dosing regimen that ensures that 90% or more of the population reach above the target C_max_ concentration of 200 µg/L [18] and stay below the mHb threshold of < 10% [31]. Since smokers have a 38% increase in CL (Table 2), smokers were simulated with a single dose of 125 mg while the nonsmokers were simulated with 100 mg of oral triapine. The simulated dosing regimen resulted in 93.9% of smokers and 90.9% of nonsmokers exceeding the target concentration and 91.4% of smokers and 92.4% of nonsmokers staying below the mHb threshold concentration (Fig. 5a).

The second aim was to evaluate the effect of variability between dosing occasions (BOV) on the percentage of the population that exceeds the mHb threshold when multiple doses are administered, as typically seen in clinical settings. BOV was evaluated on the bioavailability parameter. A population of 1000 subjects were simulated with 100 mg of oral triapine given once daily for 28 days with BOV ranging from 0% − 60% (Fig. 5b). As %BOV increased, the percentage of the population exceeding the mHb threshold also increased. The percentage of the population is shown as % increase in dose (i.e., total number of doses > 10% mHb concentration) or % increase in subject (i.e., total number of subjects with > 10% mHb concentration).

## Discussion

In this study, the first population PK/PD model was developed to characterize the relationship between triapine exposure and clinically relevant safety metrics for methemoglobinemia. The PK of triapine was best described by a two-compartment model with two-transit compartment Erlang absorption and first-order elimination, and the PD of triapine was best described by an effect compartment model. The current PK/PD model estimated all PK and PD parameters with good precision and the individual plots of the observed and predicted concentration-time profiles demonstrated that the final model adequately fits the PK and PD profiles of triapine. Overall, the final PK/PD model can be reliably utilized to inform decision-making about triapine exposure and relevant safety measures to enhance treatment success.

Triapine is linked to dose-limiting methemoglobinemia [17, 20–22, 34], making its hemoglobin iron toxicity inseparable from its efficacy. Managing methemoglobinemia is crucial because elevated concentrations can impair the blood’s ability to carry oxygen, leading to serious complications like hypoxia and organ damage [31, 35]. Thus, it can limit the safe use of triapine, ultimately affecting the efficacy and the overall treatment plan. The correlation of mHb concentrations with triapine exposure, which we present here, emphasizes the significance of optimizing triapine exposure. This study is the first to develop a quantitative model to evaluate the mHb-inducing effect of triapine, offering a systemic approach to understanding its impact on treatment outcomes.

A previous study by Kolesar et al., 2011 [32] has modeled the IV infusion triapine data using a 3-compartment model. In their study, plasma and erythrocyte triapine concentrations were sampled over a 24-hour period. Their model was able to resolve a very rapid red-cell distribution phase (Q1 = 99 L/hr) plus a slower tissue phase (Q2 = 8 L/hr) and thereby attribute much of the early decline to distribution, yielding a lower elimination clearance (CL = 25 L/hr) [32]. In contrast, the current study only had plasma data and used a 2-compartment model, yielding higher elimination clearance (CL = 51 L/hr) and a single, peripheral compartment (Q = 12 L/hr). Differences in CL estimates between the two studies are likely attributable to variations in datasets, analytical methods, and model structures. The current study employed rich PK sampling with a sensitive LC-MS/MS assay, whereas the Kolesar et al. study used a UV-based method. The LLOQ was substantially lower in the current study (3 µg/L) than in the Kolesar et al. study (0.078 µg/ml or 78 µg /L). Despite the higher LLOQ value, the Kolesar et al. study reported measurable concentrations at 24 h TAD, while concentrations at this time point were largely below the LLOQ in the current study. These differences in quantifiable terminal-phase data likely influenced estimation of the terminal elimination slope and, consequently, CL estimates. Overall, differences in analytical methodology between the two studies likely account for much of the discrepancy.

In vitro studies have shown that triapine is highly metabolized by CYP1A2 [23]. Smoking and other factors, including caffeine, cruciferous vegetables, charbroiled meats, body mass index, and oral contraceptives, can potentially impact the induction of CYP1A2 [24, 36, 37]. Studies have shown that CYP1A2 activity increases by 1.22-fold for 1–5 cigarettes, 1.47-fold for 6–10 cigarettes, 1.66-fold for 11–20 cigarettes, and 1.72-fold for > 20 cigarettes per day [38]. The increased metabolic activity due to induced CYP1A2 enzymes can lead to reduced drug efficacy, requiring higher doses to achieve the desired therapeutic effect. For instance, a meta-analysis of the interaction between smoking and the anticoagulant warfarin has shown that smoking increases warfarin clearance by 10–13% and is linked to a roughly 12% higher dosage requirement compared to nonsmokers [39]. Similarly, a meta-analysis on the impact of smoking on blood concentrations of the antipsychotic clozapine has shown that clozapine blood concentrations are significantly lower in smokers compared to nonsmokers, and a 30% dose reduction is recommended when a patient stops smoking [40]. The current model estimated effect size of smoking on CL was 0.38 with 95% confidence interval [0.19, 0.62] (Table 2), suggesting that the smokers have 38% higher clearance compared to the nonsmokers. In addition, smokers had approximately 40% lower AUC_(0−∞)_ and C_max_ estimated from noncompartmental analysis (Table S2) suggesting reduced exposure to triapine and the potential need for dosage modifications. The developed PK model in this study reduced the %CV for clearance from 33% to 29% when smoking status was included as a variable, indicating that accounting for smoking status improved the predictability of triapine clearance within the observed population [41] (Fig. S3). By effectively capturing and identifying these covariates, the developed population PK model can be utilized to facilitate more informed dosing recommendations for therapeutic decisions.

The final PK/PD model was used to simulate an optimal dosing strategy for oral triapine with minimal effect on mHb concentrations. The higher simulated oral triapine dose at 125 mg may be an optimal dosing strategy to achieve clinical benefit in smokers equivalent to that of the 100 mg dose in nonsmokers. This suggests that incorporating smoking status into dosing considerations is essential for optimizing triapine treatment and ensuring effective therapy in smokers. A prospective study with a parallel cohort for smokers and nonsmokers at their respective predicted doses with PK and PD endpoints is warranted to validate these model predictions.

In addition, the effect of BOV on the bioavailability parameter was assessed to evaluate the consistency of drug response. This was an exploratory assessment to better understand the reliability of patient responses to triapine across multiple dosing occasions, similar to what is observed in clinical settings. In 1000 simulated subjects with 100 mg dose given once daily for 28 days, with increasing %BOV, both the total number of doses and the total number of subjects who exceeded the mHb threshold increased. This suggests that ignoring BOV can lead to a distorted view of the risk profile, as it does not account for fluctuations in drug exposure that may occur due to factors such as patient adherence or physiological responses. This oversight can result in an underestimation of the actual percentage of patients exceeding the mHb threshold. Conversely, including BOV allows for a more accurate representation of the variability in drug response among patients. While it is still early in development and the true characterization of BOV remains undetermined, including BOV in future analysis may be crucial for assessing the safety of a treatment regimen.

The developed PK/PD model can be applied to inform the design of clinical trials, focusing on optimizing dose selection and safety metrics. However, there are limitations to consider: the study is not prospectively powered to assess the covariates and is exploratory due to the small sample size. Updating the model with more data will enhance its predictive capabilities and facilitate informed decision-making in future studies.

## Supplementary Information

Below is the link to the electronic supplementary material.

Supplementary Material 1

## Data Availability

The data underlying this article were provided by NCI by permission. Data will be shared on request to the corresponding author with permission of NCI.
