# Supplementary material for: Enhancing triapine treatment: strategies for dose optimization and methemoglobin level mitigation
Source: Cancer Chemother Pharmacol. 2026 May 20;96(1):55. doi: 10.1007/s00280-026-04898-6 (PMC13190473; doi:10.1007/s00280-026-04898-6)
Supplement: Supplementary file 1 — Supplementary Material 1 [file 280_2026_4898_MOESM1_ESM.pdf]

Supplementary Material:

Figure S1. Individual concentration-time curves.

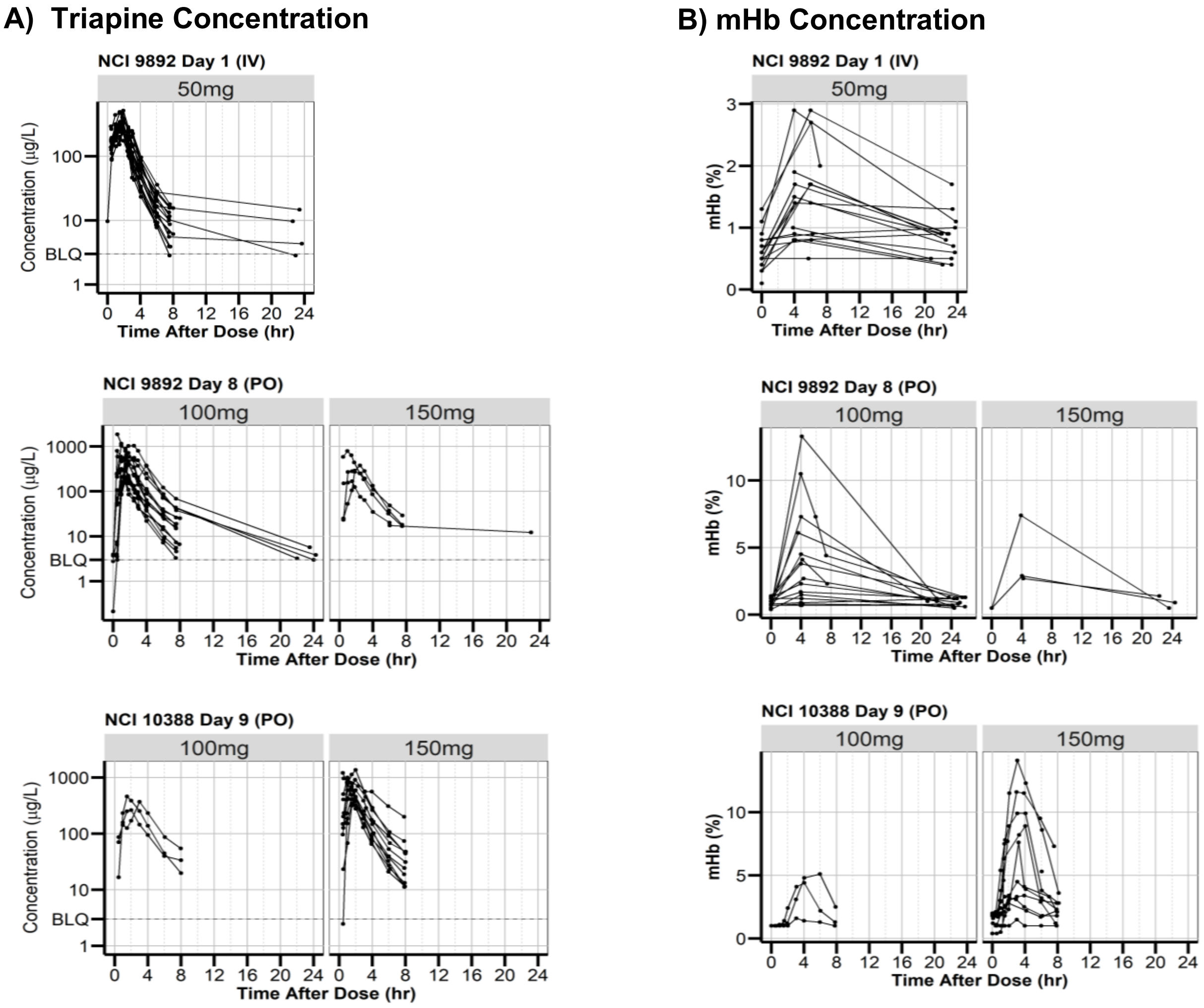

Individual concentration-time curves are plotted by study, dose, and day on a semi-log scale. **A)** Triapine concentrations. For subjects in study NCI 9892, PK samples were collected at the following time points: prior to dose, 0.5, 1, 1.5, 2, 2.5, 3, 4, 6, 8, and 24 hours after dose. At the 24-hour time point, most individuals had concentrations below the lower limit of quantification (LLOQ); hence LLOQ data point for those individuals are not shown. For subjects in study NCI 10388, PK samples were collected at the following time points: prior to dose, 0.5, 1, 1.5, 2, 3, 4, 6, and 8 hours after dose. **B)** Methemoglobin (mHb) concentrations. For subjects in study NCI 9892, PD samples were collected at the following time points: prior to dose, 4, and 24 hours after dose. For subjects in study NCI 10388, PD samples were collected at the following time points: prior to dose, 0.5, 1, 1.5, 2, 3, 4, 6, and 8 hours after dose.

Figure S2. Triapine concentration and mHb relationship shows hysteresis.

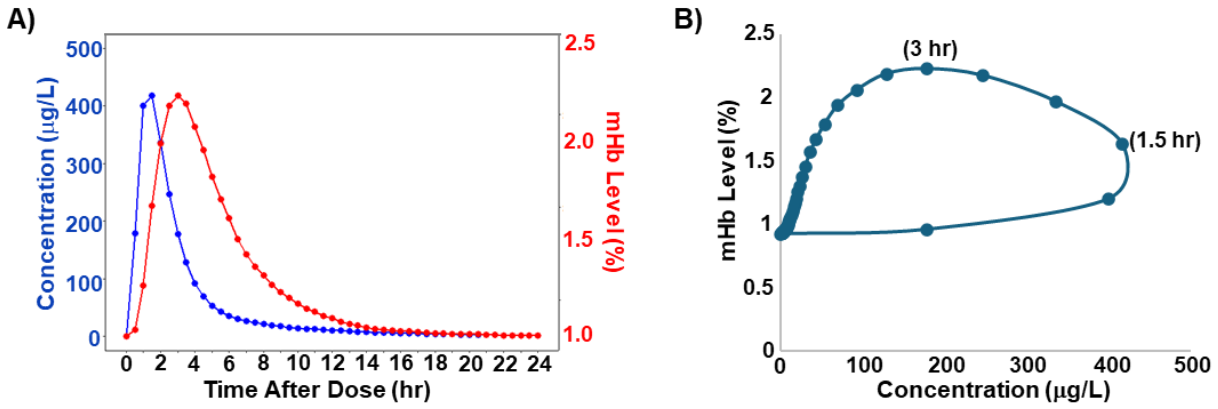

The median triapine concentrations are plotted against the median mHb concentrations from 1000 simulated datasets. Each dot represents a timepoint, spaced 30 minutes apart. **(A)** Peak concentration (blue, left Y-axis) occurs at 1.5 hours while peak mHb concentration (red, right Y-axis) occurs at 3 hours, indicating a delay in the mHb response. **(B)** The hysteresis loop is counterclockwise, suggesting that the mHb effect lags behind triapine concentration. Time points indicated in parentheses are time after dose.

Figure S3. Empirical Bayes Estimates on CL versus selected Covariates.

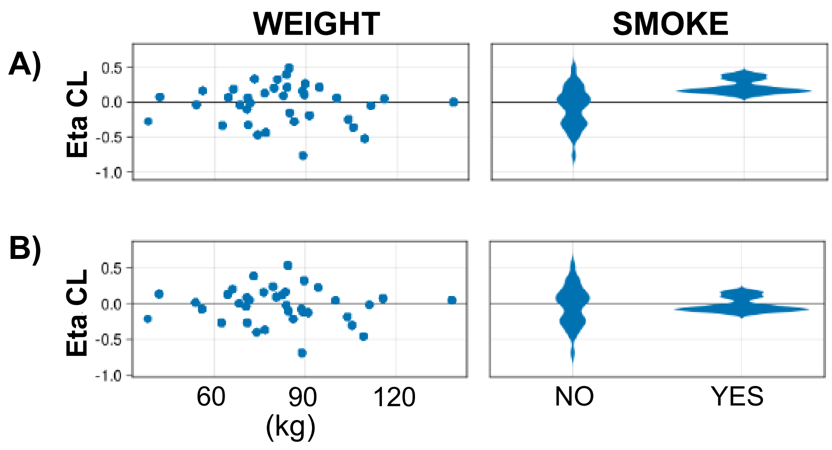

The effect of smoking reduces individual variability in clearance (CL). **(A)** When weight is the only covariate considered, the distributions of smoking status do not symmetrically center around 0. **(B)** After adjusting for the smoking status as a covariate, the distributions center around 0, indicating that smoking status explains the observed effect.

Table S1. Comparison of parameter estimates using M1 and M3 methods.

	**Laplace Estimation**	
**Parameters**	**M1**	**M3**
Ktr	3.16	2.94
CL	50.4	54.1
Vc	66.9	53.9
Q	11.9	22.3
Vp	64.0	32.1
F	0.79	0.79
Effect_smoker	0.39	0.38
Ω^2_Ktr	0.21	0.17
Ω^2_CL	0.08	0.08
Ω^2_Vc	0.05	0.08
Ω^2_F	1.34	1.38
σ^2_additive	14.8	21.5
σ^2_proportional	0.10	0.10

Parameter estimates were obtained using the M1 and M3 methods (Beal, 2001) with the Laplace estimation method. The similarity in CL estimates between the M1 and M3 methods suggests that the exclusion of LLOQ observations had minimal influence on the estimation of CL, indicating that the information contained in the censored data was not critical for characterizing drug elimination.

Abbreviations: Ktr, transit rate constant; CL, clearance; Vc, central volume of distribution; Q, intercompartmental clearance; Vp, peripheral volume of distribution; F, bioavailability; Effect_smoker, effect of smoking on CL; $\sigma_{additive}$, additive residual error; $\sigma_{proportional}$, proportional residual error; CI, confidence interval.

Table S2. Non-compartmental analysis results reported as average estimates.

Dose (mg)	C_{max} (μg/L) geomean (geosd)	$AUC_{(0-\infty)}$ (μg/L*h) geomean (geosd)
**Inf 50mg**		
Smoker (n = 6)	262 (1.2)	656 (1.1)
Nonsmoker (n = 14)	324 (1.3)	903 (1.4)
**PO 100mg**		
Smoker (n = 4)	286 (1.6)	742 (2.1)
Nonsmoker (n = 15)	544 (1.9)	1321 (1.9)
**PO 150mg**		
Smoker (n = 2)	252 (1.8)	1067 (1.2)
Nonsmoker (n = 15)	620 (1.8)	1765 (1.7)

Geomean, geometric mean; geosd, geometric standard deviation

Supplemental Methods:

All codes are in Pumas:

Methods S1: Two-compartment pop PK model with two-compartment Erlang absorption

```
mod_erlang_comb_wt_smoke = @model begin

  @param begin # defining what will be estimating/ fixed effects

    tvcl ∈ RealDomain(; lower = 0.0001)
    tvvc ∈ RealDomain(; lower = 0.0001)
    tvktr ∈ RealDomain(; lower = 0.0001)
    tvq ∈ RealDomain(lower = 0.0001)
    tvvp ∈ RealDomain(lower = 0.0001)
    tvbio ∈ RealDomain(lower = 0.0001, upper = 1.0)
    tveff_smoker ∈ RealDomain(lower = 0.001, upper=1)
    Ω²_bio ∈ RealDomain(lower = 0.0001)
    Ω²_cl ∈ RealDomain(lower = 0.0001)
    Ω²_vc ∈ RealDomain(lower = 0.0001)
    Ω²_ktr ∈ RealDomain(lower = 0.0001)
    σ²_add ∈ RealDomain(lower = 0.0001)
    σ²_prop ∈ RealDomain(lower = 0.0001)
  end

  @random begin
   ηcl ~ Normal(0, sqrt(Ω²_cl))
   ηvc ~ Normal(0, sqrt(Ω²_vc))
   ηktr ~ Normal(0, sqrt(Ω²_ktr))
   ηbio ~ Normal(logit(tvbio), Ω²_bio)
  end

  @covariates WEIGHT SMOKE_NUM SMOKE TAD

  @pre begin # how estimates will be used in dynamics block
    wtcl = (WEIGHT/70)^0.75
    wtv  = WEIGHT/70
    CL = tvcl * wtcl * (1+(SMOKE_NUM*tveff_smoker)) * exp(ηcl) # clearance
    Vc = tvvc * wtv * exp(ηvc)
    Ktr = tvktr * exp(ηktr)
    Q = tvq  * wtcl
    Vp = tvvp * wtv
  end

  @dosecontrol begin
    _bioav = logistic(ηbio)
```

```
    bioav = (; Depot = _bioav , )
  end

  @dynamics begin
      Depot' = -Ktr * Depot
      Transit1' = Ktr * Depot - Ktr * Transit1
      Transit2' = Ktr * Transit1 - Ktr * Transit2
      Central' = Ktr * Transit2 - (CL+Q)/Vc*Central + (Q/Vp)*Peripheral
      Peripheral' = (Q/Vc)*Central - (Q/Vp)*Peripheral
  end

  @derived begin
    cp = @. Central/Vc
    TRI  ~ @. Normal(cp, sqrt(((abs(cp)^2)*σ²_prop) + σ²_add))
  end

end

##############################################################################
```

Methods S2: Effect Compartment PD model:

```
mod_erlang_comb_effcmp = @model begin
  @param begin
      tvke0 ∈ RealDomain(lower=0.0001)
      tvebase ∈ RealDomain(lower=0.0001)
      tvemax ∈ RealDomain(lower=0.0001)
      tvhill ∈ RealDomain(lower=0.0001)
      Ω²_ebase ∈ RealDomain(lower = 0.0001)
      Ω²_emax ∈ RealDomain(lower = 0.0001)
      σ²_add_pd ∈ RealDomain(lower = 0.0001)
      σ²_prop_pd ∈ RealDomain(lower = 0.0001)

  end

  @random begin
    ηebase ~ Normal(0, sqrt(Ω²_ebase))
    ηemax ~ Normal(0, sqrt(Ω²_emax))
  end

  @covariates iCL iVc iQ iVp iKtr ibioav_Depot SMOKE_NUM TAD

  @pre begin
      CL = iCL
      Vc = iVc
      Ktr = iKtr
```

```
        Vp = iVp
        Q = iQ
        bioav = ibioav_Depot

        ke0   = tvke0
        ebase = tvebase*exp(ηebase)
        ec50  = 900
        hill  = tvhill
        emax  = tvemax*exp(ηemax)
    end

    @dosecontrol begin
        bioav
    end

    @dynamics begin
        Depot' = -Ktr * Depot
        Transit1' = Ktr * Depot - Ktr * Transit1
        Transit2' = Ktr * Transit1 - Ktr * Transit2
        Central' = Ktr * Transit2 - (CL+Q)/Vc*Central + (Q/Vp)*Peripheral
        Peripheral' = (Q/Vc)*Central - (Q/Vp)*Peripheral
        Ceff' = ke0*(abs(Central/Vc) - Ceff)
    end

    @vars begin
        edrug  = (emax*Ceff^hill)/(ec50^hill + Ceff^hill)
        eff_   = ebase*(1 + edrug)

    end

    @derived begin
      eff  = @. eff_
      mHb ~ @. Normal(eff, sqrt(((abs(eff)^2)*σ²_prop_pd) + σ²_add_pd))
    end
end
```
